# “What does the word loneliness mean to you?” Reflections on co-developing public engagement workshops to facilitate discussions of loneliness with older adults from ethnically diverse groups

**DOI:** 10.1186/s40900-025-00800-5

**Published:** 2025-11-13

**Authors:** Jessica Rees, Madiha Sajid, Circle Steele, Marie Croker, Wei Liu, Faith Matcham

**Affiliations:** 1 Department of Global Health and Social Medicine, King’s College London, London, UK; 2 Public Contributor, London, UK; 3 Wai Yin Society, Manchester, UK; 4 Israac Centre, Sheffield, UK; 5 Department of Engineering, King’s College London, London, UK; 6 School of Psychology, University of Sussex, Falmer, UK

**Keywords:** PPIE, Public involvement, Public engagement, Loneliness, Older adults, Ethnicity

## Abstract

**Background:**

Loneliness is a growing public health concern with known health and wellbeing consequences. The issue is often overlooked in ethnically diverse groups despite language and cultural barriers increasing the risk of social isolation. It is important to deepen our understanding of loneliness in later life from a multi-cultural perspective to design loneliness interventions that are appropriate within and across ethnic groups. In this commentary, we present a case study of our involvement activities with underrepresented ethnic groups to design and facilitate loneliness workshops. We share a joint reflection from academic and public authors on lessons learned and recommendations for future engagement activities.

**Methods:**

In three, one-off public engagement workshops, we aimed to create an inclusive and culturally appropriate space for people from ethnically diverse groups to facilitate discussion on loneliness in later life and share strategies to manage emotions. In a co-production process, a lived experience contributor worked closely with a university researcher to design the Connect and Cope workshops. Next, we collaborated with public contributors from organisations supporting older adults in the communities who facilitated discussions in the preferred language of workshop attendees. All workshops were tailored to the specific needs of each group.

**Results:**

Between June and July 2023, we engaged 46 people over the age of 65 from South Asian (*n* = 10), Chinese (*n* = 16) and Somali (*n* = 20) communities. Our Reflections are presented in three themes: 1) Co-design process, where we reflect on the value of lived experience advice in workshop design; 2) Facilitation of workshops, where we reflect on the importance of shared experience and language; and 3) Engagement with community groups, where we reflect on the role of trusted gatekeepers to support engagement.

**Conclusion:**

We highlight the benefits of a co-production approach with members of the community being engaged. For future PPIE activities involving ethnically diverse groups, we recommend utilising community organisations to act as trusted gatekeepers between universities and communities of interest. A deeper understanding of loneliness from a multi-cultural perspective is vital for informing the development of culturally appropriate interventions for loneliness reduction.

## Abstract (plain language summary)

Loneliness is very common, and people from diverse ethnic groups are often excluded from meaningful support. Stereotypes about multigenerational living create problems of ‘hidden loneliness’ in later life. Older adults from ethnically diverse groups have faced discrimination and language barriers throughout their lives. They may have felt that services have not been designed for them or that activities offered for social connection are not culturally relevant. Increasing our understanding of loneliness within ethnic groups may help address these issues. “What does the word loneliness mean to you?” This is the question we asked in the Connect and Cope workshops to begin a conversation about loneliness with older adults from three ethnically diverse groups. Between June and July 2023, we engaged 46 people over the age of 65 across the UK from South Asian (*n* = 10), Chinese (*n* = 16) and Somali (*n* = 20) communities. After the workshops, all the older adults reported knowing more about loneliness. These workshops would not have been possible without support from the communities in which we were engaging. This provided valuable insight into the specific needs of each group, creating an inclusive space in which older adults were happy to discuss their experiences of loneliness. Having a person lead the workshop in the language that the attendees felt most comfortable with was key to relationship development. Future work to engage ethnically diverse groups in sensitive discussions such as loneliness should utilise the expertise of community organisations supporting older adults at the start of projects.

## Background

In the UK and internationally, lifespan is increasing. Over the next 25 years, the proportion of people aged 85 and over is projected to double in the UK [1]. By 2050, over 2 billion people worldwide are expected to be over 60 years old [2]. Loneliness is a growing concern in a rapidly ageing society. As Age UK noted in 2018, 1.4 million older people class themselves as often lonely, and it is estimated that this number will increase to 2 million people over the age of 50 by 2026 [3]. Loneliness is also a prevalent issue in ethnically diverse groups, with language and cultural barriers increasing the risk of social isolation [4]. In the UK, 40–50% of older adults from Chinese, African, Bangladeshi and Pakistani communities have reported feeling lonely always/often, whereas the national estimates are around 10% [5].

Loneliness is defined as “a subjective, unwelcome feeling of lack or loss of companionship” associated with a “mismatch” between the quantity and quality of the social relationships we have and want [6, 7]. Although universal and typically experienced in a U-shaped curve, decreasing and increasing throughout the life course [8], loneliness is a fundamentally subjective feeling, unique to each individual [9]. Loneliness may be related to a lack of social contact (i.e. social loneliness), a lack of meaningful relationships (i.e. emotional loneliness), or a sense of separation from the world (i.e. existential loneliness) [10]. The need to broaden this definition to a multi-dimensional perspective of loneliness including an individuals’ social environment (i.e. social capital, mobility, childness, marital status) has been proposed to adopt a more holistic view of loneliness [11]. Definitions of loneliness originate from North America and Europe thus cross-cultural comparisons of loneliness have been recommended [12]. Similarities in conceptualisations of loneliness have been revealed in countries with different levels of social embeddedness (i.e. Austria, Bulgaria, Israel, Egypt, India). Definitions relate to a negative experience separate from being alone, where there is an impaired relationship between the self and the outside world [13]. However, experiences of loneliness were highly diverse both within and between cultures warranting further exploration into multi-cultural experiences of loneliness in ethnically diverse communities.

Loneliness has been associated with reduced quality of life and predicts poor physical and mental health [14, 15]. To address this public health issue, in 2018, the UK published a cross-government strategy to raise awareness of loneliness and improve support for lonely people in England [16]. However, it is important to recognise that loneliness is a culturally sensitive experience, and researchers have noted that the evidence included in strategies such as this are based predominantly on the experiences of White populations [17]. Studies have demonstrated the interaction between age, gender and culture to predict loneliness [18]. For example, compared with white people, older adults from Black and Asian communities are more likely to report having no close friends and fewer friends who live locally, demonstrating an increased risk of social isolation and loneliness [17]. Older adults from ethnically diverse groups are exposed to stressors over their life course such as discrimination [11, 19] which accumulate to negatively affect both mental and physical health [20]. In a qualitative study exploring the meaning of loneliness and social isolation from four ethnically diverse groups in New Zealand, Asian older adults focused on racial discrimination as key to their experiences of loneliness [21]. Importantly, non-white groups are reportedly more likely to feel that a service was not for them due to a feeling staff do not reflect the community in which they serve, or in some cases feeling unwelcome due to experiences of racist attitudes when accessing services [22]. Systematic discrimination and racism can impact an individual’s self-worth and feelings of belonging which contribute to feelings of loneliness [23].

Acknowledging loneliness has been identified as an important first step to addressing feelings of loneliness [24]. However, older adults from various ethnic backgrounds may be hesitant to discuss feelings of loneliness due to cultural norms which attach stigma to the topic [23]. Stigmatising views of loneliness, defined as a culturally shared belief which causes shame to those who feel lonely and make it harder to seek support, are reportedly stronger in people living in collectivist cultures [25]. Facilitating discussions with older adults on experiences of loneliness can be used to manage the stigma of the topic by framing questions in the third person [26] and enabling the identification of support needs [27]. Given the heterogeneity between different ethnic and cultural groups, inviting collaboration from diverse groups of older people themselves [21] is key to understanding the needs and experiences within such communities [5]. Patient and Public Involvement and Engagement (PPIE) is a widely used term in the UK to describe when activities are “carried out with or by members of the public, rather than to, about or for them” [28]. This is important for overcoming stereotypes that have contributed to the problem of ‘hidden loneliness’, for example, the incorrect assumption that older adults from ethnic groups living in multigenerational households are protected against loneliness [29].

However, current involvement practices are perceived by some as exclusive [30], with members of the public taking part in PPIE reportedly being from specific backgrounds where involvement is more accessible to them [31]. Previous research has highlighted tensions created from the level of skill and investment required of individuals involved in PPIE work [32]. Understanding the barriers to engagement, such as limited awareness of opportunities, the accessibility of meetings, and insufficient financial compensation, is vital to improve the inclusion of specific groups, including Black, Asian & minority ethnic groups, who continue to be underrepresented in PPIE work [33]. The need for increased inclusivity and diversity within PPIE has been highlighted in previous works [34] to make activities accessible and relevant to specific communities, improve the quality and applicability of research, and to redress existing inequalities [35]. Recommendations have been proposed to increase inclusion, namely, being community specific, using trusted figures in the community to gain access then physically going to familiar venues to develop relationships [36]. Building relationships is important to readdress power, which is typically held by academic institutions, and can lead to a lack of trust and confidence in underrepresented groups [37]. Early engagement with communities where researchers continually show how valuable feedback is acted upon has been found to build trust and increase inclusion of ethnically diverse groups in PPIE activities [35].

### Objectives

Case studies documenting PPIE in practice with ethnic minority communities are rare [35, 38]. This commentary aims to add to the evidence base of useful lessons learned from our inclusive public engagement in ageing work. To provide a case study of Involvement within a research project this Commentary will:

- Describe the process of co-production with experts by experience in the design of the workshops.
- Outline how we collaborated with voluntary organisations supporting older adults from ethnically diverse communities to facilitate each workshop.
- Present reflections from academic and public authors including lessons learnt and recommendations for future research.

The Connect and Cope workshops were a series of one-off public engagement activities hosted in Summer 2023 designed to facilitate conversations about loneliness with people from ethnically diverse groups. In the next section, we outline the context of the PPIE activity.

### Context

For a qualitative interview study exploring behaviours and indicators of loneliness in later life [9], the academic authors of this Commentary (JR, FM, WL) experienced a lack of ethnic diversity during recruitment with 90% of the sample identifying as White British (54 out of 60 participants). To address this limitation, the NHS Maudsley Biomedical Research Centre Race and Ethnicity Advisory (READ) group were consulted on recruitment strategies to engage ethnically diverse communities. The members of the READ group were from a range of different ethnic backgrounds and had an interest in mental health research and a commitment to improving the inclusivity of research. A one-hour group meeting was attended by three members. An executive summary of the project and a study poster were shared two weeks in advance of the meeting to allow time for review. Members discussed how, in some cultures, loneliness is a difficult feeling to admit. One person described loneliness as ‘nearly shameful’, as it meant the failure of family members to meet the social needs of elderly relatives. On the other hand, some cultures are recognised to embrace healthy ageing and positive attitudes towards loneliness. Moreover, the group advised that the cultural appropriateness, compassion and sensitivity of the research might need further consideration. To achieve this goal, academic authors obtained additional funding for PPIE activities to run a series of workshops with older adults from different ethnic groups to facilitate discussions about loneliness and increase understanding of loneliness from a multi-cultural perspective.

## Public involvement – developing the workshops

Public involvement is defined as work that is done with or by members of the public who have relevant experience to contribute to how activities are designed, conducted and disseminated [39]. It provides inclusive opportunities to work together, rather than research being conducted about, or for people [40]. In this commentary the term “lived experience contributor” or “public contributor” is used to describe public authors of this Commentary (MS, CS, MC) who took part in involvement activities [41], specifically the co-design and co-facilitation of loneliness workshops. This is part of a co-production approach where researchers and public contributors work together, sharing power and responsibility during PPIE activities [42]. The term “co-design” is used throughout this Commentary as a category of co-production to describe the design of workshops which positions expert by experience knowledge at the centre [43].

A personal interest in loneliness was expressed by one member of the READ panel (MS), who identified as British Pakistani. Upon receiving funding for PPIE activities, this individual was invited to collaborate as a lived experience contributor in the development of workshops to facilitate discussion of loneliness in later life. Monthly meetings from February 2023 took place between the first author (JR) and the lived experience contributor (MS) to co-design a public engagement workshop taking place in June 2023. The final workshop title was agreed as follows:

Break the silence: Connect and cope with loneliness, workshop on stories and strategies

Collaborative discussions first centred around the aim and objectives of the workshop. The first author suggested introducing the concept of loneliness by exploring definitions provided by participants in a recent interview study on the experiences of loneliness in later life [9]. The lived experience contributor specified that older adults should feel a sense of empowerment and positivity when leaving the workshop. To achieve this, the lived experience contributor decided on an activity to provide strategies for attendees to manage feelings associated with loneliness. Specific objectives of the Connect and Cope workshop included:

- To facilitate discussion on attendee definition and/or experience of loneliness in later life.
- To share strategies to reduce feelings of loneliness based on managing emotions and goal setting.
- To establish relationships with members of communities and organisations who support older adults from ethnically diverse groups.

**Fig. 1:**
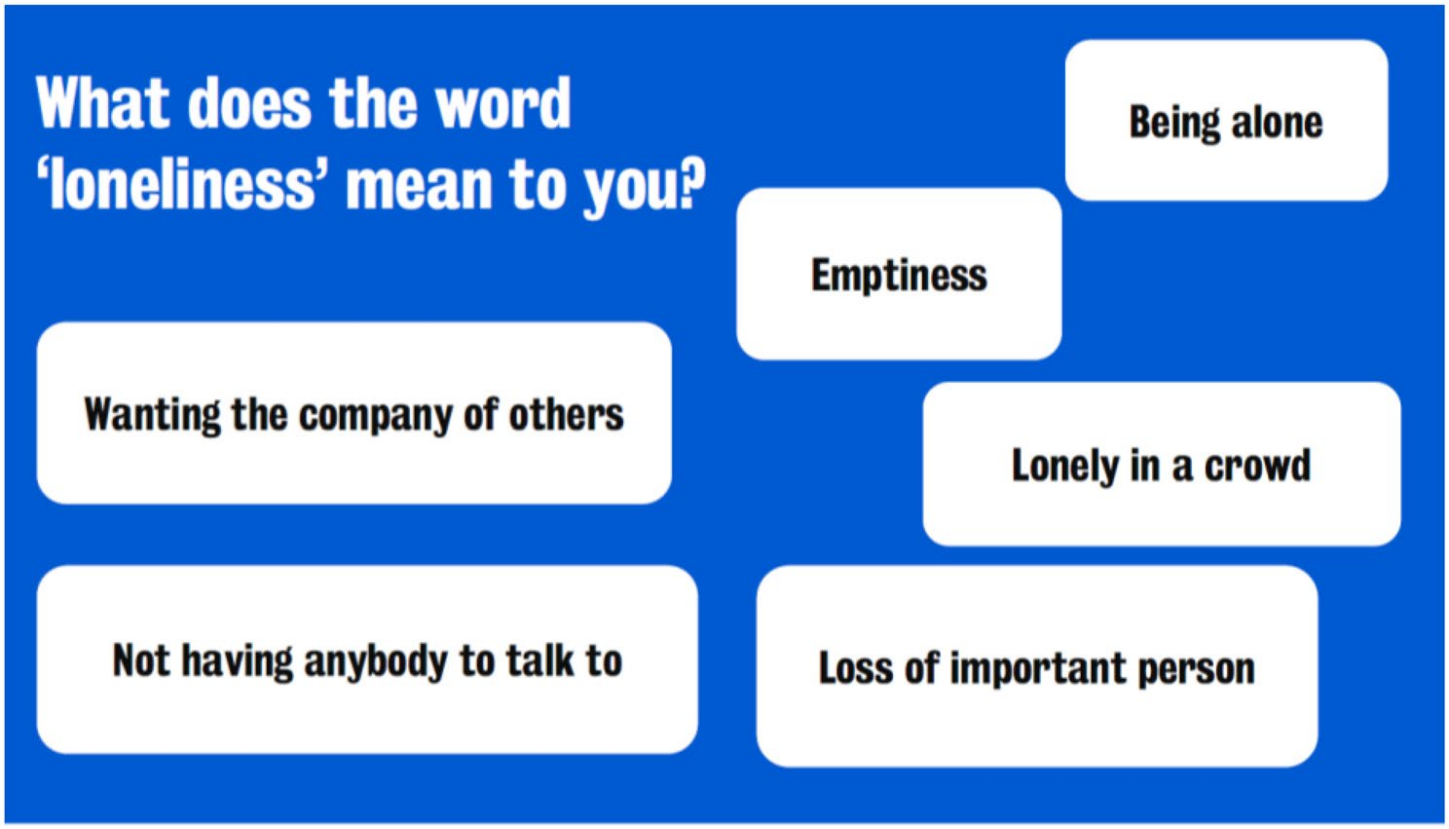
Overview of the responses from the DELONELINESS qualitative interviews

Next, the first author and lived experience contributor jointly agreed on the content of the Connect and Cope workshop. The structure began with introductions to attendees and an outline of the context for the engagement work (i.e. a predominantly white sample in research on loneliness in later life). The first author shared qualitative data of 52 older adults in response to the question “what does the word loneliness mean to you?” (see Fig. 1).

The lived experience contributor agreed that this summary of experiences would act as an anchor for discussion with workshop attendees about how they compare their experiences with the experiences of loneliness in different cultural and ethnic groups. The aim was to highlight the universal and subjective nature of loneliness to address any stigma around discussing loneliness, which may encourage attendees to speak about their own experiences.

The lived experience contributor recommended that the workshop should follow the structure of an activity followed by time for reflection before feedback. After a refreshment break, attendees shared their own reflections on feelings and experiences of loneliness. Attendees then listened to the second presentation, which focused on strategies to manage feelings of loneliness. The lived experience contributor decided on this activity which introduced the emotion wheel [44] and positive emotion, engagement, relationships, meaning, and accomplishment (PERMA) model of well-being [45]. At the end of the workshop, attendees were given a thank-you card with a £30 Love to Shop voucher and feedback forms to complete. The first author recommended such forms as an opportunity for attendees to feedback on what they liked or improvements to the workshop. This was agreed to be acceptable by the lived experience contributor. As an indicator of perceived learning, attendee knowledge of loneliness and managing emotions was captured before and after workshops. As recommended by the lived experience contributor, lunch was organised directly after the workshop to encourage further connections among attendees.

For all workshops older adults were eligible to attend if they were over the age of 65, available on the time and date of the workshop, and interested in discussing loneliness in a group setting. The lived experience contributor advised not to select participants based on self-identified experiences of loneliness as it was perceived to be a potential barrier to engagement.

In terms of roles, once the initial workshop structure was co-designed, the first author was responsible for the logistical arrangements (i.e., room booking, registering attendees, preparing slides). The lived experience contributor reviewed all the workshop material (i.e., posters, slides, and facilitation guides) prior to facilitating the workshop on the day. All the time spent by the lived experience contributor on PPIE activities (e.g., preparation, meeting attendance, workshop facilitation) was paid in line with the NIHR public contributor payment policy [46].

## Public engagement – overview of workshops

Involvement differs from Engagement, which is where information and knowledge about research are shared with the public [47]. National Coordinating Centre for Public Engagement [48] define public engagement as a two-way process involving interaction and listening with the goal of generating mutual benefit. Between June and July 2023, we engaged 46 people over the age of 65 from South Asian (*n* = 10), Chinese (*n* = 16) and Somali (*n* = 20) communities. We conducted two-hour interactive knowledge exchange workshops with older adults from three ethnically diverse groups across the UK (i.e. London, Manchester, Sheffield). To achieve an inclusive and culturally appropriate space where attendees felt able to discuss loneliness openly, we collaborated with experts by experience and community organisations supporting older adults to ensure that the workshops were tailored to the specific needs of each group. Further details are provided in the community engagement sections below.

### South Asian community engagement

A university facilitator (JR) and lived experience contributor (MS) co-facilitated a two-hour workshop with older adults from South Asian (i.e., Indian, Pakistani, and Bangladeshi) backgrounds. This workshop was hosted in a room on a university campus in Central London. The university facilitator, supported by students involved in the PPIE activity, met older adults at reception and showed attendees to the workshop location. The lived experience contributor remained in the room to welcome and chat to older adults once they arrived. Older adults in this workshop had not previously met and the lived experience contributor advised for additional time be spent at the beginning of the workshop for introductions. All attendees spoke English fluently, so no translation was required. However, as a bilingual community member, the lived experience contributor addressed attendees with culturally appropriate terms which developed rapport and trust with older adults. Following the first workshop, the university facilitator and lived experience contributor iterated the structure based on feedback, further simplifying the slides to enable easy translation in future and allowing more time for group discussion.

In terms of recruitment, older adults were invited to attend the workshop via posters shared with relevant networks (i.e. British Pakistani/Asian Foundation). The lived experience contributor supported with recruitment by sharing workshop details with these networks. Interested older adults would contact the researcher via email or telephone to register for the workshop.

The lived experience contributor shaped the workshop by providing translation in Urdu and Punjabi (which was highlighted on recruitment posters), predicting the dietary requirements of attendees (i.e. vegetarian meals), advising on attendee experience such as providing opportunities to socialise during and after the workshop, creating accessibility instructions for older adults including a picture of the workshop location and nearby travel options, and selecting culturally relevant music to make attendees feel welcome on arrival to the room.

The following two workshops were facilitated by the first author (JR) and further co-designed with public contributors (CS, MC) who work for voluntary organisations that support older adults from specific ethnic groups.

### Chinese community engagement

Founded in 1988, the Wai Yin Society is one of the largest Chinese community centres in the UK aimed at supporting and empowering Chinese individuals and their families. Academic authors were introduced to the Wai Yin Society through a contact from the University of Manchester. In March 2023, the Connect and Cope workshop was introduced to the CEO of the Wai Yin Society (CS) in a telephone meeting. Afterwards, a suitable date for the workshop was provided along with an overall budget including staff time, venue hire, interpreter fees, refreshments and lunch. The workshop was hosted in a community centre in Manchester which attendees visited frequently. The university facilitator (JR) was supported by two students who handed out resources to attendees and facilitated group discussions which were organised in smaller groups.

Workshop posters were translated by staff at the Wai Yin Society and distributed among members. Following feedback from the public contributor (CS), posters were adapted to state that interested members should contact a specific employee of the charity in person to register instead of contacting the researcher directly. A week prior to the workshop, the first author met with three staff members at the Wai Yin Society to amend the workshop structure to suit the needs of the attendees. The public contributor shaped the workshop by arranging for printouts of the presentation to be translated to Cantonese, as this was the primary language of older adults attending the workshop.

Trained community translators attended the workshop to translate content presented by the university facilitator to older adults and facilitate group discussions. The public contributor advised for discussion groups (*n* = 3) to be hosted by one university facilitator who would ask questions, and one member of staff from the Wai Yin Society, who would provide translation. Following the workshop, university facilitators met with members of staff from community organisations to provide a summary of translated feedback from workshop attendee discussions.

### Somali community engagement

Established in 1981, the Israac Centre is a registered charity providing support for communities in Shefield, with up to 60 members from the Somali community. Academic authors were introduced to the Israac Centre by colleagues at the University of Sheffield. The first author corresponded via email with the Finance and Operations Manager, and Community Development Manager (MC) of the Israac Centre. In a virtual meeting, a time and date to host the workshop was agreed.

The workshop was hosted in a community centre in Sheffield which was familiar to older adults. Attendees did not register for the workshop in advance. Older adults attending the community centre could drop-in to the workshop on the day if it was of interest. Two trained interpreters were present at the workshop who provided translations in Somali and Arabic for older adults to understand the content presented by the university facilitator.

A week prior to the workshop, the first author met with a public contributor from the Israac Centre to amend the workshop structure to suit the needs of the attendees. The public contributor shaped the workshop by advising discussion groups (*n* = 2) should be split into two groups on the basis of gender for activities in line with cultural and religious norms and recommending not to use print out materials based on the knowledge that many people aged 65 and over in this community do not read or write. Involvement of Somali attendees was not limited by the lack of printed materials as all content was discussion based.

## Reflections

In this section, academic and public authors reflect on the design and delivery of the workshops to add to the evidence base of useful lessons learnt from PPIE activities. Reflections are grouped by the following themes:

1. Co-design process, where we reflect on the value brought by experts by experience in the design of the Connect and Cope workshops.
2. Facilitation of workshops, where we reflect on the importance of shared experience and language for increasing knowledge of loneliness.
3. Engagement with community groups, where we reflect on the role of voluntary organisations supporting older adults as trusted gatekeepers.

### Co-design process

We attribute the success of the workshops to the knowledge provided by public contributors in the design of the workshop and facilitation skills to develop rapport with attendees during the workshops. Public authors highlighted the importance of co-creation at the start of any project to ensure that community voices truly shape the work and that appropriate resources are allocated. For the Connect and Cope workshops, public contributor expertise and community insight were vital. For example, workshops were organised around prayer timings and rush hour travel; appropriate words were used in messaging via emails; images in advertising were representative; interpreters, prayer rooms, and halal or vegetarian food options were made available; and simple language was used, avoiding academic terms.

When considering power dynamics, it is important to acknowledge that the public contributors involved in the Connect and Cope workshops were professional people communicating with the first author via work emails and attending meetings via Microsoft teams. The influence of the academic authors on PPIE activities was acknowledged due to power associated with holding the budget. All public contributors had individual power from their years of experience and felt confident requesting financial compensation for their contribution. When making decisions throughout the process, power defaulted to public contributors as the academic author presented an initial structure which was then shaped by advice from public contributors. All contributions were heard and acted upon. Power was shared as both academic and public authors jointly recognised the value both sides brought to the design and facilitation of workshops. This was cemented in debrief sessions following each workshop which recognised and celebrated individual contribution. The academic and public authors developed a strong relationship through the PPIE activities which enabled their voices to be integrated throughout the project.

### Facilitation of workshops

Workshop attendees valued the information provided in the workshop which increased their awareness of the issue of loneliness. When rating their knowledge of loneliness before and after each workshop all groups reported an increase after the Connect and Cope workshop. We attribute this positive outcome to the creation of an inclusive space where attendees felt comfortable sharing their own experiences of loneliness in a group setting. This enabled attendees to hear accounts of loneliness that either resonated with their own experiences or challenged their perceptions of what loneliness means. Attendees valued the opportunity to engage in group discussions to hear about different issues and speak to those in a similar position through sharing experiences.

As workshops focused on one cultural group, this enabled the sharing of specific experiences that might not have otherwise been possible. The ability to share experiences was further influenced by the workshop discussions being conducted by a facilitator from the same cultural group as the attendees. Being able to speak the same language as older adults attending the workshop was identified by public authors as the most important factor in relationship building with older adults. Overall, this helped shift the focus of workshops from the university research project to the community itself redressing power typically held by academic institutions. Language is a critical aspect to reflect on as in two workshops, university facilitators relied entirely on interpreters to translate workshop content, facilitate group discussion, and feedback on older adults’ experiences of loneliness. It is important to acknowledge that interpreters shape meaning and translations would require further consideration, especially culturally specific terminology, if collecting data on loneliness from multiple language groups. However, the aim of the Connect and Cope workshop was to facilitate discussions of loneliness, and the fact that university facilitators were excluded from group discussions due to language was an insightful shift in power dynamics.

### Engagement with community groups

Our PPIE activities had a positive outcome in terms of building relationships with community organisations who support older adults. The quote below demonstrates the reciprocal relationship between the university researchers and voluntary organisations supporting older adults:

*Working hand in hand, we have successfully produced culturally appropriate information that resonates with the Chinese older people. This accomplishment has been made possible through the close collaboration between our staff members, who possess valuable insights into the users’ situations … From the perspective of the older people involved in the project, they have expressed feeling listened to and empowered to share their voices and experiences with researchers. (CEO of Wai Yin Society)*

The Connect and Cope workshops would not have been possible without the engagement of community groups. For the Chinese and Somali workshops, voluntary organisations recruited and supported all older adults to attend the workshops. The South Asian workshop was advertised in relevant cultural networks shared by the lived experience contributor. Such a role could be defined as a trusted gatekeeper, where an individual or group controls the access of a researcher to a group of people or a particular setting.

At a dissemination event for the Connect and Cope workshops, a representative from the community organisation supporting Somali older adults (IY) spoke about how the workshops created a “springboard effect”, where members of the community felt better able to discuss loneliness among close networks. Trust was identified as an important factor in increasing the engagement of older adults from ethnically diverse groups in PPIE activities, with the proposal that introductions must be made by the community. Given the valuable contribution of community groups evidenced in the Connect and Cope workshops, public authors have suggested that PPIE activities should increase voluntary sector involvement to prevent organisations being treated only as delivery partners (Table 1).

**Table 1:** Recommendations for future work

• For future PPIE activities involving ethnically diverse groups, we recommend a co-design approach with members of the community who are being engaged to ensure an inclusive and culturally appropriate environment.
• Future studies should include members from voluntary organisations as co-applicants in project funding, an approach that builds stronger, more sustainable partnerships.
• In the future, if several workshops are being conducted, we recommend meeting ahead of each workshop to tailor the structure to the specific needs of the group.
• Public contributors who are less familiar or confident with facilitation and workshop administration may require further support for engagement.
• Consider obtaining ethical approval to enable dissemination of findings from similar workshops, acknowledging the dynamic change resulting from the formal process of informed consent.

## Discussion

In our work, we successfully engaged 46 older adults from South Asian, Chinese and Somali communities in interactive workshops about loneliness in later life. The workshops would not have been possible without the involvement of public contributors from the communities in which we were engaging. The involvement of members of the community in the design and facilitation of the workshop enabled the creation of an inclusive, culturally appropriate space for attendees to speak about topics such as loneliness and emotions. PPIE provided valuable insights into the attendee experience, which academic authors would not have thought of. These included the best time of day for workshops, simple wording of the posters, gender-specific discussion groups, and vegetarian catering options. Ensuring inclusivity in future work is imperative to address the lack of diversity in PPIE groups [34].

PPIE activities such as the Connect and Cope workshops can develop a more in-depth multicultural perspective of loneliness in later life, which is essential to identify definitions that do not inadvertently exclude different cultural groups. This is important for improving avenues for diverse communities to benefit from policies and interventions targeting loneliness. A co-production approach with people from ethnically diverse groups has been recommended to ensure loneliness interventions are tailored to individual needs and that services provide a range of accessible opportunities for social connection within and across ethnic groups [49]. Older adults were happy to speak about personal experiences of loneliness with staff from community groups they regularly attended and with peers in a language they were most comfortable with. On a practical level, translations from Chinese and Somali workshops were necessary to facilitate the transfer of knowledge from the university facilitator to workshop attendees and vice versa. Having a facilitator with inside knowledge of language and culture was also beneficial for developing a rapport with attendees and addressing challenges associated with a lack of shared language between researchers and participants [50].

Recruitment was another area in which public contributors provided vital support, sharing workshop information among relevant networks not previously known to the university team and acting as a trusted gatekeeper between older adults and researchers. Trusted gatekeepers have been previously identified as key in accessing marginalised groups in research [51]. Staff members of organisations were able to fulfil this role effectively because they were intimately familiar with the needs of older adults and thus were able to advise on elements to make the workshop as accessible as possible for attendees.

### Limitations

We acknowledge that the one-off structure of the workshops negatively impacted opportunities to collect evidence of the long-term impact of the PPIE activities, for example behaviour change, social connectedness or wellbeing. This would be interesting for future PPIE work to explore, as feedback from our community partners indicated a positive influence on ongoing loneliness discussions following the Connect and Cope workshop. The limitations of 1-off workshops for engaging in ethnically diverse communities have been reported in previous research, specifically how it ‘disregards’ the value of long-term reciprocal relationships with public contributors [31]. We recommend more specific follow-up with attendees, conducted by trusted members of the community, to further demonstrate the power of co-production work. To achieve a sustainable model of involvement, continued communication should focus on how valued feedback has been acted upon and ensuring capacity and capability of supporting organisations during the grant and planning phases of PPIE activities.

### Ethical considerations

We adhered to ‘Ethical Practice Guidelines for Public Involvement and Community Engagement’ [52] in the design and facilitation of workshops by acknowledging the potential sensitivity of loneliness as a topic. The university facilitator had experience in supporting older adults to discuss loneliness in an ethical way [9, 26] which meant being prepared to respond if an attendee became distressed. Clear guidance was also set in relation to maintaining confidentiality when sharing personal experiences in a group setting to enable the creation of a safe space.

The workshops generated interesting conversations, providing insight into a multi-cultural perspective on loneliness in later life. None of the workshops were recorded. Instead, university facilitators and public contributors met after the workshops to make collaborative notes of older adults’ discussion on loneliness which were organised into themes by the first author. In this Commentary, we did not share any discussions from the workshops due to ethical restrictions (i.e., protecting anonymity/confidentiality of attendees) related to PPIE activities. In future work, we may consider obtaining ethical approval to collect ‘data’ from similar workshops. However, we acknowledge how this change in formality (i.e., consent procedures, recorded conversations) may impact the dynamics of the workshops. Public authors of this Commentary suggested that general protocols for qualitative interview studies might inadvertently discourage participation from ethnically diverse communities. For example, people may be less willing to engage due to cultural constructs of feeling ‘ashamed’ to be ‘alone’ as that might translate to being abandoned by children or wider families.

## Conclusion

In this paper, we present an outline of the co-design process with experts by experience, community engagement with public contributors and reflections on lessons learned for future engagement work with older adults from various ethnic backgrounds. Overall, our PPIE activities were successful in creating an inclusive environment, helping attendees open up about their experiences of loneliness, and increasing knowledge of loneliness among older adults from three ethnically diverse groups across the UK. In the future, longitudinal work is needed to understand the impact of PPIE activities, utilising the continued engagement of community organisations to act as trusted gatekeepers between universities and communities of interest. This involvement has implications for health and social care practices through the identification of support needs within ethnic groups, addressing barriers to involvement and informing the development of culturally appropriate interventions for loneliness reduction.

## Data Availability

Data sharing is not applicable to this article as no datasets were generated or analysed during the current study.

## Abbreviation

PPIE: Public and Patient Involvement and Engagement
